# Head-to-Head Comparison of Novel Vaccine Technologies Comes with a Minefield of Challenges

**DOI:** 10.3390/pharmaceutics16010012

**Published:** 2023-12-20

**Authors:** Dennis Christensen

**Affiliations:** Adjuvant Systems Research & Development, Croda Pharma, 2800 Lyngby, Denmark; dennis.christensen@croda.com; Tel.: +45-26200923

**Keywords:** vaccine, adjuvant, delivery system, kinetics, germinal center, prime-boost regime

## Abstract

Modern vaccine development is having a golden period, with a variety of novel subunit technologies being introduced into clinical development in recent years. This opens the opportunity to find the best platform to use for novel vaccine antigen candidates through head-to-head comparative studies. Seldom appreciated is, however, the fact that these different technologies often do not have the same optimal antigen dose ratio, prime-boost regime and peak timepoint for measuring immunity. Instead, the preclinical studies that make the basis for platform selection use standard protocols not optimized for individual vaccines and fail to make selection on an informed basis. Here, I discuss the opportunities we have to optimize vaccine platform technologies through a better understanding of vaccine priming kinetics, the optimal antigen dose and sampling time and location.

## 1. Introduction

With the increasing numbers of vaccine platforms and vaccine delivery technologies reaching clinical development stages, there is also an increasing recognition that head-to-head comparative studies are needed, in order to select the optimal vaccine strategy for novel vaccines. Such studies are most likely to take place in the early stages of vaccine development, using small animal models [1,2,3,4]. What these studies normally do not take into consideration is that, e.g., the dosage ratio, prime-boost regime and optimal sample timing will vary from technology to technology.

In all honesty, I have myself made head-to-head studies testing different vaccine platform technologies for almost 20 years now, and only in recent years have appreciated the fact that each vaccine delivery platform needs to be used with respect for its mechanism of action when performing comparative studies. In early preclinical research, we have more or less blindly followed the standard protocols for vaccine testing with 2–3 vaccinations spaced with 2–4-week intervals, using the same antigen dose. This has been the case for comparing different platforms like vectors vs. protein subunits [5,6], different delivery systems like aluminum vs. emulsion-based adjuvants [2,7,8] and different routes like i.m. vs. i.n. [9,10,11]. Looking at the long range of evidence showing that the prime-boost strategy is not a “one-size-fits-all” solution, one could ask whether we have closed our eyes to undesirable information. It is obvious that differentiated vaccination intervals with different vaccine platforms will make our lives in the lab more challenging, but recent examples revealed during the roll-out of the COVID-19 vaccines clearly illustrate that we also need to take this factor into consideration [12,13,14,15,16,17].

COVID-19 gave vaccine development a tremendous boost, leading to licensing of several novel vaccine platforms, including novel antigen platforms, adjuvants and delivery systems [18]. The fact that all the SARS-CoV-2 virus vaccines developed during the COVID-19 pandemic used the same antigen, namely the spike protein, made it possible to benchmark the durability, magnitude and quality of the immunity asserted by different platforms.

Importantly, the scarcity of vaccines in the early period after launch also led to investigations on the effect of extending the interval of delivery of the different vaccines, to best leverage those vaccines available. The primary outcome was that the initially implemented interval of 3–6 weeks between prime and boost for the BNT162b2 and the ChAdOx1 vaccines was inferior to an extended 12-week dosing interval when it comes to the induction of spike-specific antibodies [12,15,16,17]. Interestingly, these studies, conducted in different age groups, also illustrated that the extended prime-boost regime had a beneficial effect on the T cell responses for the ChAdOx1 vaccine but not as much for the BNT162b2 vaccine, illustrating that the choice of vaccination intervals affect different vaccines and vaccine platforms differently and that the vaccination protocol has to be adapted to the individual vaccine and the desired immune correlate [13,14]. This might not matter in situations where speed overrules quality, as in the case of vaccines against SARS-CoV-2, where protective immunity from vaccination should be obtained as fast as possible and where high antibody titers are more important than the quality and long-term memory of the T and B cell responses. But in other cases, such as TB, chlamydia, MenC, HIV and malaria, the quality of B and T cell responses is more important than the speed at which immune responses can be obtained. A study on MenC vaccination from 2015 showed that when followed by a Hib-MenC-TT booster dose at 12 months, infant priming with a single MenC vaccine dose at 3 months induced a higher booster antibody response than two infant doses at 3 and 4 months of age [19], suggesting that the often applied prime-prime-boost vaccination strategy is not always the best approach. Based on the results obtained during the COVID-19 pandemic, Rodriques and Plotkin reviewed clinical trials conducted on other vaccines targeting viruses and bacteria to assess the evidence for the effect of different dosing schedules and prime-boost intervals [20]. Here, they found that the available data from both vaccines against SARS-CoV2 and against other infections are consistent in that longer intervals between the first and second doses result in better priming and higher antibody titers post-dose two, at least if the second dose is given within six months. They further reflected on vaccines varying in terms of their constituents and vaccine platform design, such as subunit protein vaccines, inactivated (killed) vaccines, vector-based vaccines or conjugate polysaccharides, all leading to different immune responses which may alter with variably timed dosing intervals [20]. In such cases, choosing the most optimal vaccination protocol might be the factor tipping the balance to obtaining vaccine-induced protection, illustrating the necessity to address dosing schedules already early in vaccine development.

## 2. Vaccine Pharmacokinetics and Immune Responses

The current dogma is that live-attenuated vaccines induce a high-quality immune response that can be difficult to attain with non-live vaccines, partially due to their facilitation of prolonged exposure of the antigens to the immune system, during the mild infection they cause, through a constrained replication. Over the last 10 years, a number of pivotal studies have in more detail shown that the kinetics of vaccine presentation can affect the immunological outcomes dramatically and that these vary from vaccine to vaccine.

In a recent study we conducted to investigate the effect of antigen association with the depot-forming adjuvant CAF01, we found that increased antigen association and thus prolonged antigen exposure correlates with the magnitude of the induced Th1 and Th17 T cell responses, but inversely with antibody induction. The latter is in line with early studies with aluminum adjuvants indicating that a too-tight binding between the antigen and adjuvant is unfavorable for antibody responses [21,22,23,24]; albeit, these classical studies with the aluminum adjuvants also showed that stronger binding to the adjuvant did not result in stronger T cell responses [21]. The data from these studies were, however, not completely consistent. In a more recent study with aluminum adjuvants, the antigens were modified to obtain different levels of electrostatic binding to Alhydrogel through site-specific introduction of multivalent phosphoserine peptide–polymer affinity tags to anchor immunogens in an oriented manner on the Alhydrogel particles [25]. This study showed, in contrast to the previous studies, that the acquisition of the antigen bound to alum drives stronger humoral responses through increased activation of BCR signaling, antigen processing and presentation. It furthermore showed that steering of alum binding can control the epitope specificity of antibody responses to an antigen, which according to the authors, might also explain the conflicting previous findings where antigens were randomly modified over the entire protein surface with alum-binding sites, which may allow the immunogen to crosslink alum particles together or become denatured upon binding, etc. In the aforementioned studies with CAF01, low B cell responses could be compensated for by increasing the antigen dose and likely relate to the prerequisite of the presentation of sufficient amounts of free antigen to the B cells being enabled by the higher antigen dose. With a higher dose of the antigen, one could thus imagine that more would be displaced from the surface of the liposomes after injection, thus leaving more antigen unprocessed by the macrophages, draining to the lymph nodes by passive diffusion and thus being made available for B cell presentation. Alternatively, more antigen is transported unprocessed to the draining lymph nodes by the innate immune cells and presented to the B cells, as suggested as a mechanism for the MF59 adjuvant [7,26]. The increased amount of antigen bound to the surface of the adjuvant might also lead to a different folding, and thus more optimal presentation of the antigen, as also suggested to matter for Alhydrogel [25]. Finally, a higher antigen dose can lead to greater production of the follicular helper T cells that govern the germinal center responses [27]. These are, however, speculations that need to be proven using in vivo tracking studies: for example, using radio- or fluorescently labeled proteins as described in [28]. It should be noted that it cannot be used as a general rule that depot formation at the site of injection is a prerequisite for a strong T cell response, clearly illustrated by GLA-SE [29] and AS01 [30], these being two non-depot-forming adjuvants both documented to induce durable Th1 responses. Their respective mechanisms of action take different paths to obtain strong Th1 responses, using squalene and QS21, respectively, to mediate strong inflammation, recruiting immune cells to the site of injection to remove the foreign matter (vaccine). This, combined with stimulation through the TLR4 receptor, induces the production of proinflammatory cytokines and chemokines that prime the APCs to mediate a Th1-type immune response [31,32]. Proper comparative studies of these adjuvant systems are, however, still missing to investigate how this affects the memory profile as well as the B and T cell epitope repertoire of the vaccine-induced immunity.

***Vaccine pharmacokinetics role in B cell induction:*** The kinetics of antigen exposure during the lifespan of the germinal centers (GCs) plays an important role in the quantity and quality of the antibody responses [27,33,34,35]. Tam et al. thus showed in very laborious kinetic studies in mice that prolonged and increasing vaccine exposure during germinal center induction leads to enhanced GC B cell differentiation and antigen-specific antibody production [34]. These results were later confirmed in non-human primates, the study also showing that the prolonged antigen exposure led to an increased antibody diversity [33].

**Figure 1:**
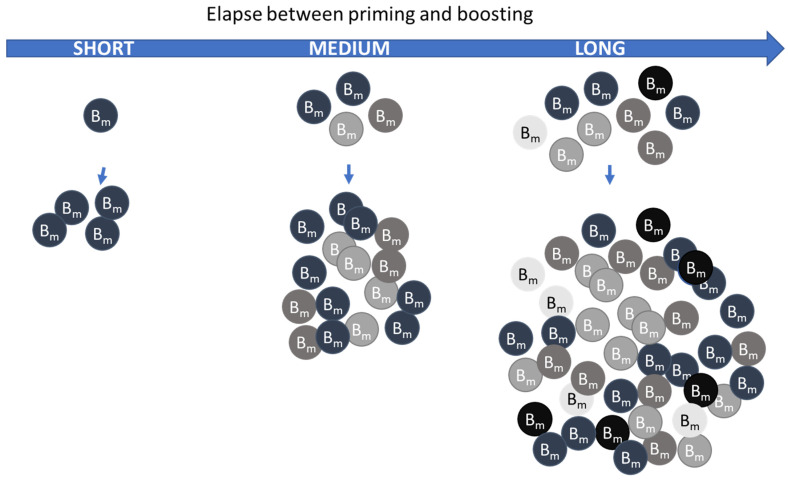
Illustration on how elapse between priming and boosting can affect memory B cell repertoire ready to be boosted. The different gray color shades of the memory B cells illustrate different clones. If the duration is too short, only those B cells with highest affinity to the antigen will be activated and expand after boosting (left side vertical arrow). Longer duration will leverage more B cell clones with a higher-than-baseline affinity and thus result in a higher-avidity antibody response with a broader range of different B cell clones, and in addition a higher number of memory B-cells ready to be boosted (middle and right side vertical arrow).

It is furthermore well recognized that vaccine-induced high-avidity antibodies are obtained by leaving memory B cells to rest several months after germinal center reaction. The longer duration from germinal center collapse until vaccine boosting will thus leverage more B cell clones with a higher-than-baseline affinity and thus a higher-avidity antibody response [36] (Figure 1). In addition, as also illustrated in Figure 1, during this extended period of time, the memory B cells will also proliferate and thus expand the numbers of cells ready for reactivation.

In summary, high-quality humoral responses are best obtained through continuous (preferably elevating) antigen availability over the course of the GC lifetime, followed by a resting period—in humans, of typically 4–6 months—to expand and increase the avidity of the memory B cells before a booster vaccination. Is this adhered to in our preclinical models used for the selection of the best vaccine candidate?

As previously mentioned, vaccine testing in preclinical studies in animals—no matter the species—is typically conducted with 2–3 vaccinations spaced with 2–4-week intervals. The pitfall of this practice is that one unwillingly incorporates an advantage bias toward those vaccines with fast vaccine kinetics and renounces those vaccines which are more prone to prolonged antigen presentation kinetics to show their full potential, as described above, thus missing out on opportunities for higher-quality vaccination. This can be illustrated by recent studies conducted comparing vaccine adjuvants with depot-forming and fast drainage properties. Knudsen et al. compared five different adjuvants combined with three different vaccine candidates against influenza, tuberculosis and chlamydia [2]. The adjuvants tested were Alhydrogel, the two squalene-based adjuvants MF59 and GLA-SE and the two cationic adjuvants IC31 and CAF01. A standard vaccination protocol with three immunizations with a three-week interval and immunological evaluation two weeks after each immunization was used for all antigen/adjuvant combinations. The overall trend was that the emulsion-derived adjuvants mediated both faster and stronger antibody responses compared to the depot-forming cationic adjuvants. This observation for the squalene-based adjuvants is in line with a study conducted by Calabro et al. who, using fluorescent forms of MF59 and ovalbumin antigen, found that the recruited innate immune cells, arriving to the site of injection shortly after immunization, take up both the adjuvant and antigen to transport them to the draining lymph nodes, peaking within the first 24 h [7]. In a study comparing early molecular signatures in the blood and draining the LN of vaccines encompassing CAF01, IC31, GLA-SE and Alum, it was also clear that not much happened in the first week with the depot-forming adjuvants (CAF01 and IC31), where most of the immune activity is likely to happen at the site of injection, compared to the non-depot-forming adjuvant (GLA-SE), and that germinal center formation for GLA-SE is observed already after 72 h (likely before), whereas the depot-forming adjuvants only showed a trend toward GC formation after 1–2 weeks [8]. A follow-up study confirmed this observation and showed that the germinal center formation is substantially delayed when using depot-forming adjuvants such as CAF01 compared with the squalene emulsions, the latter peaking after a couple of weeks, whereas CAF01-induced germinal centers only peak after six weeks or maybe even longer [37]. With the previously mentioned knowledge that booster immunization should optimally come after germinal center collapse [36], it is apparent that the prime/boost regime in the mentioned adjuvant comparison study was maybe acceptable for the squalene emulsions, but at best suboptimal for CAF01, since the germinal centers has not even peaked in size yet after three weeks. Along with investigating the germinal center kinetics, we also showed, in contrast to previous studies [2], that the antibody response magnitude of the CAF01-formulated vaccine superseded that of the squalene emulsion, but with a delay correlating with the germinal center kinetics [37].

***Vaccine pharmacokinetics role in T cell induction:*** The helper T cell function comes in many shapes, spanning effector, effector memory, central memory, follicular helper, resident memory and regulatory T cells. In general terms, the effector T cells die within days of reaching this stage of activation, whereas the effector memory T cells can live for years and are easily and quickly reactivated, but have a reduced ability to proliferate compared to the central memory cells, which can furthermore persist lifelong but are only slowly reactivated. The resident memory T cells, in opposition to the central memory T cells, migrate into tissues such as the mucosa, skin and certain organs and reside there, in order to be rapidly reactivated upon local encounters with cognate antigen, and thus drive a fast local adaptive immune response. The follicular helper T cells are pivotal for activating and driving the maturation of B cells presenting the cognate antigen on the MHC in the GCs, whereas the role of inducible regulatory T cells, which are the regulatory T cells of interest from a vaccination point of view, is to ensure that the immune system does not overreact and creates more harm than good. The original dogma was that the destiny of T cells was determined at a very early stage of priming, maybe even before, but it is now recognized that there is an enormous plasticity and that, e.g., follicular T cells can turn into memory T cells and vice versa. It is, however, still the assumption that T cells, when reaching the effector stage, do not have the ability to turn into central memory or effector memory T cells.

For vaccination purposes, the induction of a good balance between central and effector memory cells is therefore most often preferred. The main determinant for the induction of memory T cells is the duration of antigen exposure during priming, whereas the amount of antigen exerts more influence on the degree of activation. A high bolus dose of antigen with a short durability will thus induce a strong short-lived effector T cell response, whereas the other extreme, with a low but prolonged antigen exposure, will mediate a strong central memory response. The type of T helper response (Th1, Th2, Th17, Treg, etc.) is determined by the co-stimulatory signaling during priming more than the duration of antigen exposure. Much of the correlations mentioned here are unfortunately more theoretically founded than proven in reality, and more studies need to be made to fully understand the relation between antigen kinetics and T cell responses.

## 3. What to Think about When Designing Comparative Vaccine Technology Studies

There is thus plenty of documentation illustrating how important it is to consider how the different vaccine technologies affect the pharmacokinetics of the antigen exposure to the immune system, how this can affect the immunological outcome and how to compensate for potential unwanted effects, such as the absence of B or T cell responses. It is thus evident that the kinetics of antigen presentation after vaccination have a major impact on both the quality and magnitude of the adaptive immunity obtained after vaccination. The big question is whether we take this aspect into consideration in the preclinical models used for the selection of the best vaccine candidates. The short answer is no!

First, in order to give each vaccine technology the most optimal conditions, we have to accept that we cannot use the same prime-boost regime for all technologies, and that studies should be designed with the possibility of varying the spacing between the prime and boost, as well as the timepoint for sample collection, adapted to the individual technology. In the end, it must be an objective of the head-to-head studies to test each technology at its best for obtaining the requested immune correlates. This will without doubt make the study design much more cumbersome but the alternative—to continue using the condensed standard regimes—will in many cases result in the deselection of the better candidates.

A natural consequence of this is that more detailed studies need to be undertaken on the individual vaccine technologies before entering into comparative studies. The knowledge required to design the optimal vaccination protocols for individual technologies includes:

1. **Vaccine priming kinetics** of the germinal centers in the draining lymph nodes. This is important for the determination of the optimal window for booster immunizations, especially when it comes to B cell boosting.
2. **Vaccine antigen dose**. The optimal dose of an antigen, whether protein-, peptide- or nucleic-acid-based, varies from antigen to antigen and (unfortunately) from species to species. Most important, however, is that the optimum antigen dose is not the same for all vaccine technologies, and for some technologies, it has a major impact on the T/B cell induction balance, as illustrated with CAF01 by Wørzner et al. [38], meaning that antigen dose optimization should be linked to the expected correlates of protection for the vaccine.
3. **Sampling timepoint.** With different pharmacokinetic windows for the different vaccine technologies, it is also clear that the optimal timeframe for test sampling will vary between technologies. These timepoints may also vary depending on the requested immune correlates; hence, it is important to make a thorough immune kinetic mapping of the B and T cell responses for each individual technology.
4. **Sampling sites.** Different immune profiles not only lead to different timepoints for sample selection but also different optimal sampling sites. Strong effector T cell and plasma cell responses are best measured in the blood, whereas central memory B and T cell responses are better measured in the primary and secondary lymph nodes and spleen. Furthermore, unless a study is designed for investigating the effector immune responses for the individual vaccine technologies at their peak timepoint, it might be necessary to compare immune responses in different compartments.

**Figure 2:**
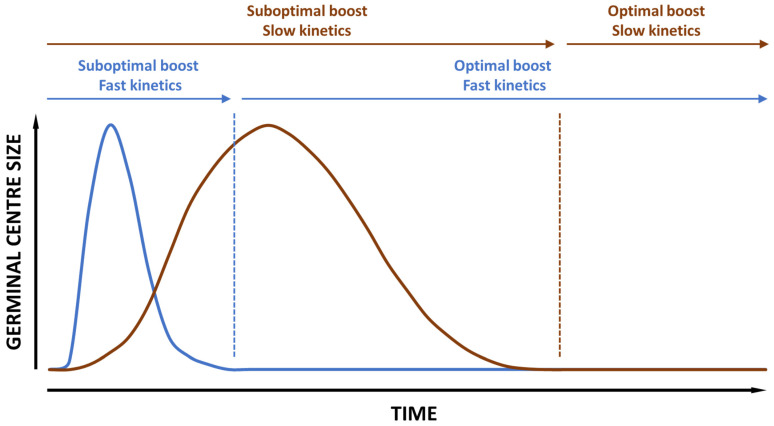
Illustrates how using the same vaccination regime for a fast (blue) and a slow (brown) GC kinetics vaccine will position the immune system to react to the booster vaccination. If the booster vaccination is adjusted to a fast kinetics vaccine technology, boosting of the slow kinetics technology will potentially happen on top of the existing germinal center, which is suboptimal. If, on the other hand, booster vaccination is conducted after germinal center collapse of the slow kinetics vaccination, the fast kinetics vaccination might have had time to settle with a stronger memory B cell response, making comparison difficult also in this scenario.

When this information is in place, the next challenge will be how to design the vaccine technology comparison studies. In the end, they must be compared according to their individual best performance. Imagine comparing a fast-acting vaccine with a slow-acting vaccine in mice, using a classical two immunizations with a four-week interval vaccination regime and sampling two weeks later. Likely, the second vaccination for the fast-acting vaccine will act as a booster vaccine following a germinal center collapse, whereas the second vaccination for the slow-acting vaccine will rather act as a second priming on top of an existing germinal center, thus prolonging its lifespan by giving access to a new reservoir of antigen (see Figure 2). These two scenarios will mediate not only the fact that the magnitude of the immune responses is different for the two vaccines, but also that the active immune sites and the measurable immune fingerprints are different for the two vaccine technologies at the time of sampling, only because of the design of experiment.

The most optimal scenario would therefore, in my opinion, be to design comparative experiments where the first sampling timepoint is adjusted to the peak responses of the individual vaccine technologies. Consequently, the timepoints for the prime and booster vaccinations will not be the same for the different vaccine technologies, where vaccination with slow-kinetics technologies might start months before that of fast-kinetics technologies.

## 4. Future Directions

With the expected increase in comparative studies on different vaccine technologies that we will see in the future, it is clear that the field must exert effort to make the most optimal out of each technology and not seek compromises to fit all technologies into one framework. But first, technology providers should acknowledge their own technologies and invest in understanding the mechanisms of action of their products, in order to advise on the best practice for their use. This will increase the investments needed to develop new technologies, but the cost is still insignificant compared to the value lost on suboptimal use. It is therefore important to invest in understanding the mechanisms of action and innate and adaptive immune induction kinetics and eventually defining the optimal prime-boost regimes for novel vaccine platforms very early in the development of novel vaccine technologies. It is appreciated that this is a major task and can seem a barrier too high. My advice would thus be, instead of the one-dimensional focus on the adaptive immune correlates, such as the B and T cell responses, to start with understanding the immune priming kinetics and mechanisms for the activation of the innate immune system, which eventually lead to the adaptive immune responses. With this knowledge, it will be possible to model and deduct many of the most important questions related to, for example, the optimal prime-boost regime, and many resources can be saved.

## Data Availability

The data presented in this study are available in this article.
